# An Analysis of the Willingness to the COVID-19 Vaccine Booster Shots among Urban Employees: Evidence from a Megacity H in Eastern China

**DOI:** 10.3390/ijerph19042300

**Published:** 2022-02-17

**Authors:** Tianzhen Hu, Li Li, Chuanxue Lin, Zikun Yang, Cheng Chow, Zhipeng Lu, Chen You

**Affiliations:** 1 School of Public Affairs, Zhejiang University, Hangzhou 310058, China; swbhtz@hz.cn (T.H.); lichs@zju.edu.cn (L.L.); 12022048@zju.edu.cn (Z.L.); youchen@zju.edu.cn (C.Y.); 2 General Office of the CPC Hangzhou Municipal Committee, Hangzhou 310020, China; 3 Center of Comparative Political Economy, Renmin University of China, Beijing 100872, China; 4 Department of Politics and International Studies (POLIS), University of Cambridge, Cambridge CB3 9DT, UK; 5 Department of Social Work and Social Administration, University of Hongkong, Hongkong 999077, China; chowcc@connect.hku.hk

**Keywords:** COVID-19, vaccine, vaccine willingness, vaccine booster shots, urban employees

## Abstract

Many studies have shown that urban workers may have a higher acceptance rate of coronavirus disease (COVID-19) vaccine uptake compared to their rural counterparts. As Omicron spreads globally, the COVID-19 booster vaccination has been acknowledged as the primary strategy against this variant. In this study, we identify factors related to the willingness of workers in megacities to take the vaccine booster shots and their main reasons accounting for their booster willingness. This research survey was conducted in megacity H in eastern China, and a total of 1227 employees from different industries were interviewed. The study at hand examines the relationship between various characteristics (including both economic and non-economic factors) of urban employees and their intention/desire to accept the COVID-19 booster shoots. The survey results show that some characteristics, namely work organization, vaccine knowledge, and social network, affect their intention to take COVID-19 vaccine booster shots. Urban employees with a strong work organization, a high degree of vaccine knowledge, and a dense social capital are more likely to receive booster injections than other employees. Therefore, work organization, vaccine knowledge, and social networks provide fundamental entry points for designing enhanced injection strategies to increase the acceptance of COVID-19 vaccines among employees in megacities.

## 1. Introduction

Within two years from 2019 to 2021, the coronavirus disease (COVID-19) pandemic swept through the entire world, evolving into one of the global public health emergencies with the most severe influence [1,2,3]. As of 20 December 2021, the number of confirmed cases of COVID-19 on the global scale has exceeded 274 million, including more than five million deaths [4]. Despite the Omicron variant’s lightning speed of infection, the spread of the vaccine has reduced deaths from COVID-19 in many countries [5,6]. It is widely acknowledged by academia that enhancing vaccination of booster doses constitutes a core strategy to contain the continuous spread of the COVID-19 pandemic [7]. In terms of fighting the Omicron variant, most of the high-income countries recommend their citizens to receive booster shots.

Vaccine acceptance rates refer to ratios of qualified individuals who take certain vaccines within a particular time range [8]. Although most states initiated public vaccination at the beginning of 2021, acceptance rates vary drastically following regional economic development (i.e., urban and rural areas; low-income and high-income countries). According to data published by the World Health Organization (WHO), by the time of 5 November 2021, the starting date of this research, only 41.6% of the global population have been vaccinated. Resistance and rejection of COVID-19 vaccines, together with vaccine hesitancy towards booster shots, are still prevalent in many areas [9,10,11]. Even in China, which is widely acknowledged as one of the countries with the smoothest vaccination programs [6], the coverage rate of booster vaccines was yet to exceed 10% of the total population as of 20 December 2021, while the National Health Commission of the People’s Republic of China (NHC) reported that 84% of citizens have completed their vaccinations by this date [12]. Many Chinese remain hesitant about taking booster shots, and widespread posts on social media indicate that the public is still wary of the necessity of getting the second dose and booster shots [13]. However, there are not many studies and investigations on the willingness to vaccine boosters. 

In view of this background, a large number of studies try to demonstrate the public’s hesitation or willingness to vaccination and to discover significant causations between vaccine attitudes and regional disparity [14,15,16]. A series of scholarships along this path elucidate the high degree of heterogeneity of vaccine willingness across each region under the influence of gender, age, political economy, traditional culture, vaccine knowledge, social networks, and other factors [10]. For instance, studies on attitudes towards COVID-19 vaccines within the global range find that among general adults, females and younger populations are groups with less willingness to accept COVID-19 vaccines. However, in comparably more affluent rural areas in Australia, the young female groups demonstrate a high level of enthusiasm towards vaccinations [17,18,19,20]. In addition, relative studies observe a relatively lower rate of COVID-19 vaccines acceptance among urban residents in the Middle East, Eastern Europe, and Russia, whereas their East Asian counterparts exhibit a higher acceptance rate, owing this disparity mainly to social capitals, a consequential factor [21]. In a study of megacities, scholars realized that urban workers’ and employees’ protection of themselves and others constitutes the primary reason for vaccine willingness, and concerns about severe side effects and safety of vaccines are tested as essential causes for refusal [22]. Other surveys found that factors attributed to urban dwellers’ hesitation included a number of socioeconomic reasons [10], personal preference and risk appetite, knowledge about COVID-19 prevention, and medical measures [21], and others, all relevant to vaccine willingness. 

Despite previous studies on megacities residents’ willingness to accept vaccination, scholars have yet to discuss the willingness of booster vaccines. This study puts a large number of economic and non-economic factors into a model for systematic analysis and finds the answer to the research question: which factors have a decisive influence on the intensified vaccination of the residents of megacities? Our paper extrapolates the vaccine situations in a megacity H in eastern China through a large-scale regional survey. In alignment with the existing studies on vaccine willingness, we focus on various factors, including both economic and non-economic components. The latter contains personal characteristics, work organizations, vaccine knowledge, medical knowledge (of booster), and social networks. The comprehensiveness of this set of variables makes it quite compatible for the survey at hand. As our results elaborate, vaccine willingness or hesitancy is attributed to some but not all these factors. 

The result of our statistical analysis demonstrates the following tendency. Some social and occupational characteristics indeed have an impact on workers’ desire to receive COVID-19 vaccine booster shots. Urban employees with higher organizational level jobs, better knowledge of vaccines, and denser social network are more willing to accept booster injections than regular workers in the cities. Consequently, work organizations, vaccine knowledge, and social networks provide fundamental entry points to design enhanced injection strategies in order to improve the acceptance rate of COVID-19 booster vaccines among employees in megacities. 

## 2. Materials and Methods

### 2.1. Sample and Setting

In March 2021, China began to open vaccination to all citizens. The vaccination is divided into two rounds, with an interval of 8 weeks between the two rounds. In September 2021, various provinces in China began to arrange booster shots, but they were officially promoted from October to December. Our research was carried out from October to December. 

The subject area of this study is megacity H in Zhejiang Province, China. Adjacent to Shanghai, H is a well-known experimental Chinese city for new and high technology, whose Internet industry and technological research and development industry are of the highest rank nationally. According to public information, the resident population of H City is 11, 936, 010, of which men account for 52.08% and women account for 47.92%. In 2020, the annual fiscal revenue of megacity H will be 385.42 billion yuan, and the disposable income of the city’s residents will reach 61, 879 yuan. At the end of 2020, City H achieved a regional GDP of 1610.6 billion yuan, of which the tertiary industry increased by 1095.9 billion yuan, accounting for 68.1%. In the composition of GDP, the core industries of the digital economy account for 26.6%. With reference to China’s definition standards for megacities, we prefer to define city H as megacity H. Therefore, these characteristics lead to easier access to technology and digital economy practitioners, as well as high-quality talents working in these industries, when conducting the survey. 

This research depends on the anonymous, self-designed, and structured regional online questionnaire survey conducted through the platform of a research firm, Wenjuan.com (https://www.wenjuan.com (accessed on 15 November 2021), in November 2021 in megacity H. The dataset of this firm covers 41 different industries in megacity H. Questionnaires emphasize anonymity and privacy protection and allow participants to exit and withdraw whenever they find themselves uncomfortable. During the COVID-19 pandemic, the method of circulating online questionnaires has obtained widespread application in epidemiology, public health management, medical sociology, and other relevant fields, because it is capable of obtaining structured social and behavioral data about the pandemic when fieldworks are no longer available [23,24,25].

Applying the convenience sampling method, this survey recruits employees from the megacity H to enter the first stage and starts with the quantitative cross-sectional study [26]. Online questionaries are circulated through the channel of social media, targeting workers from all industries that reside in megacity H, and expanding the scope of coverage through snowball sampling. Participants of the online survey can choose to indicate their interests in taking part in in-depth interviews during the second stage. These candidates are drawn by purposive sampling to ensure the diversity of interviewees selected. Both methods collect informed consent forms electronically and undertake data protection and privacy measures to secure participants’ confidentiality. 

Online questionaries used for this research endeavor to evaluate the following four points: (1) urban employees’ attitudes towards the booster shot of COVID-19 vaccines and willingness towards receiving enhanced vaccine injections in megacity H; (2) economic characteristics of urban workers in megacity H, including industry, occupation, income, and others; (3) non-economic characteristics of urban workers in megacity H, including medical knowledge about vaccine, social network, work organizations, etc.; (4) demographical characteristics of urban workers in megacity H, including age, sex, educational level, and politic countenance. Consequently, population data captured from each survey participant consist of age, sex, educational level, etc. Economic characteristics contain industry, occupation, monthly income, and others. Non-economic characteristics are constituted by medical knowledge of vaccination, social network, work organizations, and others. Besides, data on the question of whether subjects survey has received (at least) one shot of vaccine is used to assist in selecting samples suitable for further analyses. 

### 2.2. Data Collection

From 20 October to 10 December 2021, we collected confidential answers to online questionnaires within eight weeks. A trial test was conducted with groups representing 5% of the total sample size before formally releasing questionnaires to the public. This study assumes a 90% confidence interval and a 5% margin of error, and the research team recovered all test samples. On the basis of the trial test, the research group issued 1397 confidential online questionnaires. After excluding the incomplete questionnaires, the final effective sample size was 1227, and the questionnaire recovery rate was 87.8%.

Within this research period, governments of both Zhejiang Province and megacity H declared partial regions within H as zones of emergency status from 25 October to 2 November, and from 6 December to 21 December 2021. Therefore, during the primary time of research, megacity H remains under phases of strict pandemic controls.

This research adopts the SPSS 28.0 edition (IBM Corp, Armonk, NY, USA) for statistical analysis. While describing the relations between urban employees’ vaccine booster shots willingness in megacity H and economic and non-economic factors, it applies the frequency formula of descriptive statistics of SPSS. This research also utilizes SPSS factor analysis, correlation analysis, independent sample *t*-test, and other methods to analyze the aforementioned relation between megacity H’s workers’ willingness to receive booster vaccine and their economic and non-economic characteristics.

## 3. Results

### 3.1. Demographic Characteristics

The total sample size of questionnaires distributed in this study is 1397, with 1227 people (87.8%) having received COVID-19 vaccination before this research. Among them, 1227 answer sheets fully complete all the questions set in this study. Therefore, these 1227 questionnaires are valid samples for this study and are used to further analyze the willingness of urban workers towards booster shoots of the COVID-19 vaccine. In the overall sample, some groups have never been vaccinated against COVID-19, which provides a favorable control for the follow-up to ensure that the sample is sufficiently representative. Detailed demographic characteristics are exhibited by Table 1 (18–29, 471 people, 38.8%; 30–39, 442 people, 36.0%; 40–49, 207 people, 16.9%). Most of the interviews are middle-aged and young people. Within the sample, 471 individuals fall under the age range of 18–29, counting for 38.8% of the total sample. There are 442 individuals within the age range of 30–39 and constitute 36.0%. There are 207 individuals that fall under the age range of 40–49, counting for 16.9% of the total sample. This result fulfills/corresponds to the age distribution of urban employees in China [27,28]. Among them, 628 are males who count for 51.2%, and 599 are females that count for 48.8%. Within the sample, 652 people (53.1% of the total sample) have accomplished university education, and 398 individuals (32.4%) have obtained a postgraduate degree (masters and above). In terms of political countenance, 544 individuals (44.3%) are members of the Chinese Communist Party (CCP), whereas people with no party affiliation count for 609 individuals, or 49.63% of the total sample. As for occupational characteristics, there are 688 enterprise employees (56.1%), 172 staff in government-affiliated public institutions (14.0%), and 141 civil servants (11.5%). When it comes to industry, 313 individuals (25.5%) belong to the Internet industry (including Internet research and development, operation, design, etc.), 186 individuals (15.2%) work for the government and social organizations, and 136 individuals (11.10%) make their livings at the industry of technology, scientific research, and development. There are 398 people (32.4%) that earn 5000–10,001 yuan per month, while 552 individuals acquire a monthly income of 10,000–30,001 yuan.

### 3.2. Correlation Analysis: Factors Influencing Booster Vaccination

The outcome variable of this study is the respondent’s willingness to uptake COVID-19 vaccinate booster shoots. It is directly measured through three questions. For example, one of the questions is “ Will you get a booster shot of the COVID-19 vaccine in the near future?” In particular, these three questions with distinctive angles to reflect the willingness of vaccine booster shots are: Is there is a willingness to be vaccinated? How long will you be vaccinated? Will you persuade friends to take the vaccine booster shots together? The three questions above all use the 5-point Likert scale, have passed the factor analysis, and are aggregated into one variable, which is the willingness to inoculate the vaccine booster shots we discussed. Through the analysis of the outcome variables, we found that most urban employees expressed positive acceptance (737 people, 60.1%), followed by disapproval (372 people, 30.3%) and uncertainty (118 people, 9.6%). Most of the interviewees stated that they had completed the COVID-19 vaccination (1091 people, 88.9%), followed by no vaccination (106 people, 8.6%), and finally completion of the first time of vaccination (30 people, 2.4%).

Table 2 reports the main results of the questionnaire survey on the acceptance of vaccine booster shot.

Based on the above, we conducted a correlation analysis on the relationship between a variety of independent variables and dependent variables (employees’ willingness to accept vaccine booster shots in megacity H) and came to the following conclusions: First, the willingness to vaccinate booster is related to gender, and males seem to be more inclined to get vaccinated (R = −0.077, *p* < 0.05); Second, the willingness to vaccinate booster is related to the political countenance. Communist Party of China members (including probationary party members) are more inclined to receive vaccination (r = −0.109, *p* < 0.01); Third, the willingness to vaccinate booster is related to occupation. Civil servants, staff in government-affiliated public institutions, and employees of enterprises are more willing to be vaccinated (r = −0.120, *p* < 0.01); Fourth, the willingness to vaccine booster is related to the current vaccination situation (the first two shots). Respondents who have already received two shots demonstrate a stronger willingness to vaccinate (r = −0.359, *p* < 0.01); Fifth, the willingness to vaccinate booster is related to the respondent’s attitude towards the efficacy of the booster vaccine. Respondents who recognize the efficacy of booster are more willing to vaccination (r = 0.173, *p* < 0.01); Sixth, the willingness to vaccinate booster is related to the respondent’s mastery of vaccine knowledge. The more knowledge of vaccine booster shots obtains by the respondent, the stronger the willingness of vaccination (r = 0.386, *p* < 0.01); Seventh, the willingness to be vaccinated is related to the social network scale of the respondent. The larger the network scale of the respondent, the more likely they are to be vaccinated (r = 0.122, *p* < 0.01).

Table 3 reports the correlation analysis results of the questionnaire survey on the acceptance of booster vaccines.

In the next step, we need to perform an inter-group difference test (independent sample *t*-test) on the above correlation conclusions to evaluate the relationship between the outcome variable (willingness to vaccinate the COVID-19 vaccine booster shots) and each independent variable.

### 3.3. T-Test

The control situation of the t-test is based on the willingness to vaccinate booster shots (T1) as the boundary, which is divided into three groups “willing/unsure/unwilling” of which the complete control is the uncertain group, and the remaining two groups are control groups. We classify various economic or non-economic characteristics into three categories, namely personal characteristics (including job and occupation characteristics), vaccine medical knowledge, and social capital.

Firstly, we obtained the test results of differences between groups of personal characteristics and willingness to inoculate vaccine booster shots. First, the willingness to vaccinate is related to gender. Men seem to be more inclined to booster vaccinate (verified), t (willing–unwilling) = −2.374, *p* = 0.018. Second, the willingness to inoculate is related to the political countenance. Party members are more inclined to inoculate (verified), t (willing–unwilling) = −3.334, *p* = 0.001. Third, the vaccination willingness is related to occupation. Civil servants, staff in government-affiliated public institutions and employees of the enterprise are more willing to vaccination (verified), t (willing–unwilling) = −3.282, *p* = 0.001. Fourth, the vaccination willingness is related to the current personal vaccination situation (the first two shots). The respondents who have been vaccinated with two shots have stronger vaccination willingness (verified), t (willing–unwilling) = −8.682, *p* < 0.000. Fifth, the vaccination willingness is related to the respondent’s attitude towards the efficacy of the vaccine booster shots. The more the respondent recognizes the efficacy of the booster vaccine, the stronger the willingness of vaccination (verified), t (willing–unwilling) = 5.728, *p* < 0.000. Table 4 reports the results of the analysis of the *t*-test results related to personal characteristics and willingness to vaccinate booster shots.

Secondly, we calculated the t-test results of vaccine booster shots medical knowledge and vaccine booster shots vaccination willingness. Table 5 reports the results of the analysis of the results of differences between the groups on the medical knowledge of vaccines booster and the willingness to inoculate vaccine booster shots. The test results of the degree of mastery of vaccines booster knowledge show that first and foremost, the vaccination willingness is related to the degree of the respondent’s mastery of vaccine booster shots knowledge. According to the result, we infer that the more the respondents understand the vaccine booster shots knowledge, the stronger the willingness to vaccinate booster they demonstrate. However, this conclusion still needs to be supported by more evidence such as data from relevant experiments. The four questions for measuring the vaccine booster shots knowledge of respondents showed large differences between the control groups, and the *p*-values were all less than 0.001. Second, the data analysis found that due to the well-publicized vaccine and vaccine booster shots knowledge in the country and H city where the respondent is located, the respondent did not score high on only one of the four sets of test questions (question 4). However, the overall error rate of this question is low (15.6%). Third, the above analysis demonstrates that the respondent’s understanding of vaccine information through various channels can help increase their willingness to booster vaccinate. The *t*-test results of the four groups of test questions support this result, as seen in Table 5.

Finally, we analyzed the test results of the differences between the groups of social capital and the willingness to vaccinate booster shots. Here, we use the network scale, network apex, network difference, the relationship with leadership (RWL), the relationship with management (RWM), the relationship with scientific, and technical personnel (RWS) to measure the level of social capital of the respondents [29,30]. In the sample of this study, the respondents’ network scale, network apex, network difference, and RWL are aggregated into one variable, and this factor has an explanatory rate of 46.138%. Thus, the elements above are four explanations for the social capital factor. Table 6 reports the analysis of *t*-test results of the social capital and the willingness to vaccinate booster shots. To ensure the accuracy of the analysis results, we also reported the test result of the differences between the groups of the remaining two factors-RWM and RWS.

The results of the *t*-test of social capital are as follows. First, the willingness to vaccinate booster shots is related to the social network scale of the respondent. The larger the network scale of the respondent, the more likely they are to be vaccinated (partially supported). Data analysis found that the size of the network will affect the people who are still hesitating, but this effect is not linear (as the respondents who are unwilling to inoculate have a larger network). Second, the willingness to vaccination is related to the relationship between the respondent and the leadership. When the respondent’s social network has leadership ties, they are more inclined to accept vaccinate (partially supported). Data analysis found that the bond with the leadership will also affect the people who are still hesitating, but this impact is not linear (as the interviewees who are unwilling to inoculate also have a certain degree of leadership bond). Third, according to the social capital variables obtained after factor analysis and aggregation, the *t*-test found that the social capital of the respondents will have a certain impact on their vaccination, and this impact only exists in the choice of vaccination or uncertain vaccination. Fourth, the above analysis indicates that the respondent’s social network and social capital will have an impact on the respondent’s willingness to vaccinate booster shots, but this impact is not a purely linear relationship.

## 4. Discussion

It is widely believed that COVID-19 vaccines have significant benefits for the community, and many people have strong confidence in the effectiveness of vaccines in immunization and disease control. A survey conducted by CDC shows that if the patient is completely vaccinated with the COVID-19 mRNA vaccine, its effective rate has been proved to be 94% [31]. However, there are few related investigations on the willingness to vaccinate booster shots. While the Omicron variant is spreading worldwide, the inoculation of the COVID-19 vaccine booster shots is generally considered as the primary strategy against the new virus strains [32,33]. This paper fills in the lacuna in the research on the willingness to vaccine booster shots for responding COVID-19 crisis. This study found that, under the influence of the Omicron variant, the employees in City H attach great importance to the vaccine booster. For example, the proportion of employees who are willing to participate in COVID-19 vaccine booster is as high as 60.1%, although there is a gap between the willingness to vaccinate booster shots and the willingness to vaccination (in other studies, the willingness to vaccination in China and the United States is as high as more than 80%) [34,35]. These results on megacity H regarding the COVID-19 vaccine booster provide a blueprint for megacities to better formulate effective COVID-19 vaccination policies.

Besides, this study shows a significant positive correlation between personal characteristics (including job and occupation characteristics) and the willingness to vaccinate booster shots in the megacity. Among them, the political countenance, occupation, current situation of vaccination, and the attitude towards the efficacy of the vaccine booster shots are related to the willingness to vaccinate booster shots. In a specific Chinese context, Party members are in a highly organized party organization, have a good sense of social policy, and keep relatively synchronized with government policies [36]. Therefore, party members are more inclined to vaccinate booster shots and converge with the government’s policy of promoting vaccination booster shots. On the one hand, civil servants, and staff in government-affiliated public institutions are dedicated to public affairs and have a higher willingness to provide public services [37,38], making them more inclined to accept the vaccinate booster. On the other hand, Chinese enterprises, like the government and public institutions, have a highly organized capacity. The leadership often promotes the benefits of vaccine and vaccine booster shots to members of the organization, rendering the employees more inclined to vaccination. In addition, from the perspective of monthly income, since vaccination in China is free, in accordance with previous research conclusions, there is a positive correlation between vaccination willingness and income. The low vaccination cost is likely to strengthen the individual’s vaccination willingness [39,40,41]. However, in this study, employees show the opposite tendency. The higher income obtained by the groups, the less willing they become to vaccinate the booster shot, indicating that there is a negative correlation between income and willingness to vaccinate the booster shot. We believe that after the first two vaccination actions, whether these respondents have completed the vaccination or not, at present, there is no obvious evidence that the two shots previously vaccinated have significant effects, which indirectly leads to the high-income groups choosing to hesitation. As high-income groups have more vital action ability than low-income groups, they can better guarantee themselves to enjoy better medical services, thus reducing their anxiety and sense of urgency to rush to be vaccinated with booster shots. Perhaps they are also looking forward to more information showing the effectiveness or ineffectiveness of vaccine booster shots.

Secondly, there is a positive correlation between the medical knowledge of vaccine booster shots and the willingness to vaccinate booster shots. Before this study, existing scholarships were more concerned about the relationship between general vaccine knowledge and the willingness to vaccination, leaving medical knowledge of vaccine booster shots underexamined. However, the public’s attitude and perception towards vaccine booster shots and vaccines are likely to be divergent [32]. The four groups of test questions we designed are all related to the knowledge of vaccine booster shots, and the four groups of test questions all supported the significant relationship between them both. Therefore, it is evident to demonstrate that the government and other public organizations should strengthen the publicity of urban employees’ knowledge about vaccine booster shots, which will help to enhance their willingness to inoculate them.

Finally, the study found a significant positive correlation between social capital and the willingness to vaccinate with booster shots. First, the willingness is related to the social network scale of the respondents. The larger the social network scale, the more the vaccinators tend to vaccinate booster shots. This may be because megacity employees believe that vaccine booster shots can protect not only themselves but also their families and friends. This discovery shows that the network scale may play an important role in COVID-19 vaccination activities in the living environment of urban employees because communities with dense social network scales can better comply with preventive measures [42]. Second, when there is a close relationship between leadership and management in the social network of the respondents, they are more inclined to vaccinate with booster shots. We hypothesize that urban employees are afraid that COVID-19 will spread from themselves to leaders and managers, which will break up this social relationship. However, this hypothesis still needs further testing. Third, the research on the correlation between social capital and the willingness to vaccinate booster shots basically elucidates that megacity employees are also willing to receive COVID-19 vaccines to protect the community or other people in the company. Since in previous studies related to social capital, scholarships are likely to pay more attention to other preventive measures unrelated to vaccination [43,44], this discovery further improved the relevant research on social capital and COVID-19 prevention measures.

## 5. Conclusions

Based on a sample size of 1227 employees from various industries, this study tested various variables with a significant relationship between the willingness to vaccinate booster shots through the Internet questionnaire survey of City H, a megacity in eastern mainland China. This study provides further support for exploring the relationship between economic and non-economic factors and the willingness to vaccination booster shots of employees in megacities in China. Although there have been previous studies on the COVID-19 vaccination willingness of employees in megacities, few scholars have carried out discussions on the willingness to vaccinate with COVID-19 booster shots. This study found that some factors have a decisive impact on the willingness to vaccinate booster shots of employees in megacities. It shows that different factors have different effects: personal characteristics (including job and occupation characteristics), vaccine knowledge of booster shots, and social capital have a positive correlation and significant effect on the willingness to vaccinate booster shots of employees in megacity H. In contrast, the income level has a significant negative correlation with the willingness to vaccinate booster shots. In addition, we acknowledge that there are some data flaws that deserve attention. First of all, the survey sample is composed of employees in megacity H. As the city has developed a high-tech industry and is located on the southeast coast of China, the income level and education level of the employees are high, and they are mainly young and middle-aged. At the same time, the way of using online questionnaires affects the characteristics of the respondents to a certain extent. For instance, high-quality talents and people who are good at using the Internet can more easily access our questionnaires. Secondly, this research is mainly based on the Chinese context. Therefore, the main conclusion of our research includes the option of political countenance, and it is found that the political countenance has different effects on the willingness of urban employees to obtain COVID-19 vaccine booster shots. This particularity applies to the background of this research. The situation may vary depending on the cultural environment. Finally, although this survey has cross-sectional and regionally representative, compared with the huge population of megacity H, the sample size is relatively small. Furthermore, in this study, megacity H was directly affected by COVID-19, and some of the samples were collected during the government-mandated partial lockdown. Therefore, this paper cannot completely avoid the potential risk of social desirability. Future studies need to break through this limitation and make more representative national-level studies of cross-country studies. In fact, this paper clarifies the misunderstandings of western countries about China’s administrative compulsory vaccination. The sample data shows that Chinese urban residents are more collective, rather than subject to mandatory booster shots.

## Figures and Tables

**Table 1:** Distribution of demographic characteristics of the sample (*n* = 1227).

Category	Variable	Number	% (*n*)
Age	Under 18	5	0.40%
	18–29	471	38.80%
	30–39	442	36.00%
	40–49	207	16.90%
	50–59	78	6.40%
	Beyond 60	24	2.00%
Gender	Male	628	51.20%
	Female	599	48.80%
Education	Junior high school and below	14	1.10%
	High school, higher vocational or tertiary qualifications	50	4.10%
	College and related qualifications	113	9.20%
	Bachelor’s degree	652	53.10%
	Postgraduate and above	398	32.40%
Politic countenance	CCP member	544	44.34%
	Other democratic parties’ member	30	2.44%
	Personnel with no party affiliation	44	3.59%
	Nonpartisan	609	49.63%
Occupation	Civil servants	141	11.50%
	Staff in government-affiliated public institution	172	14.00%
	Enterprise employee	688	56.10%
	proprietorship	36	2.90%
	Teacher	54	4.40%
	Doctor	18	1.50%
	Self-employed	80	6.50%
	Other occupations	38	3.10%
Industry	Public administration, social security and social organization	186	15.20%
	Technology, scientific research and development	186	15.20%
	Agriculture, forestry, husbandry, fishery	18	1.50%
	Electricity	24	2.00%
	Energy, petroleum and chemical industry	10	0.80%
	Mining	7	0.60%
	Internet (including Internet research and development, operation, design, etc.)	313	25.50%
	Journalism and communication	34	2.80%
	Finance	91	7.40%
	Medical and nursing	72	5.90%
	Education	102	8.30%
	Social organization and social work	36	2.90%
	Business (including self-employed business, catering, etc.)	6	4.60%
	Logistics	10	0.80%
	Others	132	10.80%
Monthly income	3000 yuan and below	39	3.20%
	3001–5000 yuan	134	10.90%
	5001–10, 000 yuan	398	32.40%
	10, 001–30, 000 yuan	552	45.00%
	More than 30, 000 yuan	104	8.50%

**Table 2:** Summarized findings on the acceptance of vaccine booster shots among employees in H city.

Variable		Number
COVID-19 vaccine booster shot acceptance		
Do you intend to be booster vaccinated against COVID-19?	Yes	737 (60.1%)
	Uncertain	118 (9.6%)
	No	372 (30.3%)
Have you completed the COVID-19 vaccination?	Yes	1091 (88.9%)
	Only finish the first time	106 (8.6%)
	No	30 (2.4%)
Do you think the vaccine booster shot is effective against COVID-19 (especially Omicron)?	Yes	773 (63.0%)
	Uncertain	416 (33.9%)
	No	38 (3.1%)

**Table 3:** Correlation analysis of acceptance of vaccine booster shots.

□	1	2	3	4	5	6	7	8	9	10	11	12	13	14	15
Age	1	□	□	□	□	□	□	□	□	□	□	□	□	□	□
2. Gender	0.077 **	1													
3. Education	−0.223 **	−0.015	1												
4. Politic countenance	−0.198 **	0.028	−0.337 **	1											
5. Occupation	0.02	0.129 **	−0.138 **	0.262 **	1										
6. Industry	0.05	0.162 **	−0.224 **	0.242 **	0.412 **	1									
7. Income (monthly)	0.058 *	−0.126 **	0.260 **	−0.118 **	−0.158 **	−0.159 **	1								
8. Vaccination status	0.03	0.100 **	0.003	0.053	0.108 **	0.01	0.047	1							
9. Attitude towards booster’s curative effect	0.077 **	0.103 **	−0.135 **	0.014	0.066 *	0.072 *	−0.118 **	−0.028	1						
10. Knowledge of vaccines	0.029	−0.017	0.041	−0.085 **	−0.107 **	−0.032	0.062 *	−0.163 **	0.041	1					
11. Log-network scale	0.122 **	−0.068 *	−0.035	−0.137 **	−0.083 **	−0.006	0.150 **	−0.043	0.075 **	0.059 *	1				
12. Log-network apex	0.118 **	−0.019	−0.029	−0.096 **	−0.083 **	−0.042	0.004	0.006	−0.014	0.047	0.175 **	1			
13. Log-network difference	0.085 **	−0.078 **	0.043	−0.105 **	−0.024	−0.025	0.03	−0.02	−0.008	0.006	.337 **	0.514 **	1		
14. EXP-RWL ^a^	0.095 **	−0.052	0.105 **	−0.187 **	−0.149 **	−0.132**	0.055	−0.031	0.031	0.022	0.170 **	0.176 **	0.248 **	1	
15. Willingness to vaccinate	0.042	−0.099 **	−0.067*	−0.109 **	−0.120 **	−0.024	−0.063 *	−0.359 **	0.173 **	0.386 **	0.122 **	0.035	0.02	0.016	1

^a^ EXP-RWL = exp(relationship with leadership); ** At the 0.01 level (sig), the correlation is significant; * At the 0.05 level (sig), the correlation is significant; *N* = 1227.

**Table 4:** *t*-test on personal characteristics (occupational characteristics) and willingness to the vaccine booster shots.

Characteristic	No. of Responses	Survey Sample	Willingness to Receive a COVID-19 Vaccine?			*p*-Value	*p*-Value
		(*N* = 1227)	Yes (*n* = 737)	Not sure (*n* = 118)	No (*n* = 372)	Yes vs. Not Sure	Yes vs. No
**What is your gender?**	□	□	□	□	□	0.084	**0.018**
Male	1227	733(59.7)	463 (63.2)	64 (8.7)	206 (28.1)		
Female	1227	494(40.3)	274 (55.5)	54 (10.9)	166 (33.6)	□	□
**What is your political countenance?**	1227	□	□	□	□	0.364	**0.01**
CCP member	1227	544 (44.3)	358 (65.8)	48 (8.8)	138 (25.4)		
Other democratic parties’ member	1227	30 (2.4)	14 (46.7)	8 (26.7)	8 (26.7)		
Personnel with no party affiliation	1227	44 (3.6)	18 (40.9)	4 (9.1)	22 (50)		
Nonpartisan	1227	609 (49.7)	347 (57)	58 (9.5)	204 (33.5)	□	□
**What is your occupation?**	1227	□	□	□	□	0.176	**0.001**
Civil servants	1227	141 (11.5)	101 (71.63)	14 (9.93)	26 (18.44)		
Staff in government-affiliated public institution	1227	172 (14.0)	106 (61.63)	14 (8.14)	52 (30.23)		
Enterprise employee	1227	688 (56.1)	418 (60.76)	68 (9.88)	202 (29.36)		
Proprietorship	1227	36 (2.9)	20 (55.6)	0 (0)	16 (44.4)		
Teacher	1227	54 (4.4)	26 (48.1)	2 (3.7)	26 (48.1)		
Doctor	1227	18 (1.5)	6 (33.3)	4 (22.2)	8 (44.4)		
Self-employed	1227	80 (6.5)	36 (45.0)	14 (17.5)	30 (37.5)		
Other occupations	1227	38 (3.1)	24 (63.2)	2 (5.3)	12 (31.6)	□	□
**Have you completed the COVID-19 vaccination?**	1227	□	□	□	□	0.184	**<0.001**
Yes	1227	1091 (88.9)	709 (65.0)	106 (9.7)	276 (25.3)		
Only finished the first time	1227	30 (2.4)	2 (6.7)	4 (13.3)	24 (80.0)		
No	1227	106 (8.6)	26 (24.5)	8 (7.5)	72 (67.9)	□	□
**Regarding the view of the vaccine booster shots in the treatment of COVID-19?**	1227	□	□	□	□	**<0.001**	**<0.001**
Not effective	1227	38 (3.1)	8 (21.1)	4 (10.5)	26 (68.4)		
Relatively effective	1227	416 (33.9)	216 (51.9)	56 (13.5)	144 (34.6)		
Very effective	1227	773 (63.0)	513 (66.4)	58 (7.5)	202 (26.1)	□	□

**Table 5:** *t*-test on vaccine medical knowledge and willingness to the vaccine booster shots.

Characteristic	No. of Responses	Survey Sample	Willingness to Receive a COVID-19 Vaccine?			*p*-Value	*p*-Value
		(*N* = 1227)	Yes (*n* = 737)	Not Sure (*n* = 118)	No (*n* = 372)	Yes vs. Not Sure	Yes vs. No
**The antibody formed by the vaccine has a certain half-life. When it drops to a certain level, can the antibody level be increased by injecting the vaccine (booster)?**						0.146	**<0.001**
Yes (right answer)	1227	961 (78.3)	649 (67.5)	96 (10.0)	216 (22.5)		
No	1227	54 (4.4)	8 (14.8)	0 (0)	46 (85.2)		
Not Sure	1227	212 (17.3)	80 (37.7)	22 (10.4)	110 (51.9)		
**There are memory cells in the immune system?**						0.426	**<0.001**
Yes (right answer)	1227	957 (78.0)	621 (64.9)	94 (9.8)	242 (25.3)		
No	1227	28 (2.3)	10 (35.7)	0 (0)	18 (64.3)		
Not Sure	1227	242 (19.7)	106 (43.8)	24 (9.9)	112 (46.3)		
**Is the booster of the COVID-19 vaccine an inactivated vaccine?**						**0.003**	**<0.001**
Yes (right answer)	1227	809 (66.0)	551 (68.1)	72 (8.9)	186 (23.0)		
No	1227	58 (4.7)	18 (31.0)	8 (13.8)	32 (55.2)		
Not Sure	1227	360 (29.3)	168 (46.6)	38 (10.6)	154 (42.8)		
**The booster of the COVID-19 vaccine for young children may cause depression and other mental illnesses?**						0.159	**<0.001**
Yes (right answer)	1227	192 (13.2)	74 (38.5)	14 (7.3)	74 (38.5)		
No	1227	348 (28.3)	250 (71.8)	32 (9.2)	66 (19.0)		
Not Sure	1227	717 (58.5)	413 (57.6)	72 (10.0)	232 (32.4)		

**Table 6:** *t*-test on social capital and willingness to the vaccine booster shots.

Characteristic	No. of Responses	Survey Sample (*n* = 1227)	Willingness to Receive a COVID-19 Vaccine?			*p*-Value Yes vs. Not Sure	*p*-Value Yes vs. No
			Yes (*n* = 737)	Not Sure (*n* = 118)	No (*n* = 372)		
			Mean (SD)				
**Network scale-logarithm**	1227	1227 (100)	1.51 (0.29)	1.41 (0.30)	1.48 (0.30)	0.018	0.309
**Network apex-logarithm**	1227	1227 (100)	1.68 (0.58)	1.65 (0.59)	1.63 (0.65)	0.745	0.382
**Network difference-logarithm**	1227	1227 (100)	0.45 (0.39)	0.39 (0.37)	0.43 (0.36)	0.34	0.58
**Interviewee’s relationship with leadership-EXP (RWL)**	1227	1227 (100)	2.12 (0.82)	2.15 (0.81)	2.12 (0.82)	0.001	0.952
**Interviewee’s relationship with management-EXP** **(RWM)**	1227	1227 (100)	1.25 (0.60)	1.35 (0.70)	1.28 (0.63)	0.289	0.583
**The relationship with scientific and technical personnel-EXP** **(RWS)**	1227	1227 (100)	1.18 (0.52)	1.15 (0.48)	1.12 (0.44)	0.666	0.178
**Social capital-logarithm**	1227	1227 (100)	0.14 (0.13)	0.09 (0.14)	0.13 (0.13)	0.009	0.488

## Data Availability

Data used in this study cannot be made publicly available for ethical reasons. Public availability of data would compromise the confidentiality and privacy of participants.

## References

[1] Xiao Y., Torok M.E. (2020). Taking the right measures to control COVID-19. Lancet Infect. Dis..

[2] Mathieu E., Ritchie H., Ortiz-Ospina E., Roser M., Hasell J., Appel C., Giattino C. (2021). A global database of COVID-19 vaccinations. Nat. Hum. Behav..

[3] Randolph H.E., Barreiro L.B. (2020). Herd immunity: Understanding COVID-19. Immunity.

[4] World Health Organization General WHO Coronavirus Disease (COVID-19) Dashboard. https://covid19.who.int/.

[5] Haas E.J., Angulo F.J., McLaughlin J.M., Anis E., Singer S.R., Khan F., Brooks N., Smaja M., Mircus G., Pan K. (2021). Impact and effectiveness of mRNA BNT162b2 vaccine against SARS-CoV-2 infections and COVID-19 cases, hospitalisations, and deaths following a nationwide vaccination campaign in Israel: An observational study using national surveillance data. Lancet.

[6] Wake A.D. (2021). The willingness to receive COVID-19 Vaccine and its associated factors: “Vaccination refusal could prolong the war of this pandemic”—A systematic review. Risk Manag. Health Policy.

[7] Crocker-Buque T., Edelstein M., Mounier-Jack S. (2017). Interventions to reduce inequalities in vaccine uptake in children and adolescents aged <19 years: A systematic review. J. Epidemiol. Commun. Health.

[8] Sallam M., Dababseh D., Yaseen A., Al-Haidar A., Ababneh N., Bakri F., Mahafzah A. (2020). Conspiracy beliefs are associated with lower knowledge and higher anxiety levels regarding COVID-19 among students at the University of Jordan. Int. J. Environ. Res. Public Health.

[9] Chou W.-Y.S., Budenz A. (2020). Considering emotion in COVID-19 vaccine communication: Addressing vaccine hesitancy and fostering vaccine confidence. Health Commun..

[10] Troiano G., Nardi A. (2021). Vaccine hesitancy in the era of COVID-19. J. Public Health.

[11] Dror A.A., Eisenbach N., Taiber S., Morozov N.G., Mizrachi M., Zigron A., Srouji S., Sela E. (2020). Vaccine hesitancy: The next challenge in the fight against COVID-19. Eur. J. Epidemiol..

[12] Mallapaty S. China’s COVID Vaccines Are Going Global—But Questions Remain. https://www.nature.com/articles/d41586-021-01146-0.

[13] National Health Commission of the People’s Republic of China. http://www.nhc.gov.cn/jkj/s7915/202112/9b0efe79ee6b4b8286c5e745541c8b2e.shtml.

[14] Allington D., Duffy B., Wessely S., Dhavan N., Rubin J. (2020). Health-protective behaviour, social media usage and conspiracy belief during the COVID-19 public health emergency. Psychol. Med..

[15] Sallam M. (2021). COVID-19 Vaccine Hesitancy Worldwide: A Concise Systematic Review of Vaccine Acceptance Rates. Vaccines.

[16] Al-Amer R., Maneze D., Everett B., Montayre J., Villarosa A.R., Dwekat E., Salamonson Y. (2021). COVID-19 vaccination intention in the first year of the pandemic: A systematic review. J. Clin. Nurs..

[17] Freeman D., Loe B.S., Yu L.-M., Freeman J., Chadwick A., Vaccari C., Shanyinde M., Harris V., Waite F., Rosebrock L. (2021). Effects of different types of written vaccination information on COVID-19 vaccine hesitancy in the UK (OCEANS-III): A single-blind, parallel-group, randomized controlled trial. Lancet Public Health.

[18] Prickett K.C., Habibi H., Carr P.A. (2021). COVID-19 Vaccine Hesitancy and Acceptance in a Cohort of Diverse New Zealanders. Lancet Reg. Health West. Pac..

[19] Schwarzinger M., Watson V., Arwidson P., Alla F., Luchini S. (2021). (COVID-19 vaccine hesitancy in a representative working-age population in France: A survey experiment based on vaccine characteristics. Lancet Public Health.

[20] Carter J., Rutherford S., Borkoles E. (2022). COVID-19 Vaccine Uptake among Younger Women in Rural Australia. Vaccines.

[21] Trueblood J.S., Sussman A.B., O’Leary D. (2021). The Role of Risk Preferences in Responses to Messaging About COVID-19 Vaccine Take-Up. Soc. Psychol. Personal. Sci..

[22] Wang K., Wong E.L.Y., Ho K.-F., Cheung A.W.L., Yau P.S.Y., Dong D., Wong S.Y.S., Yeoh E.-K. (2021). Change of willingness to accept COVID-19 vaccine and reasons of vaccine hesitancy of working people at different waves of local epidemic in Hong Kong, China: Repeated cross-sectional surveys. Vaccines.

[23] Hur J., Chang M.C. (2020). Usefulness of an online preliminary questionnaire under the COVID-19 pandemic. J. Med. Syst..

[24] Ahmad A.R., Murad H.R. (2020). The impact of social media on panic during the COVID-19 pandemic in Iraqi Kurdistan: Online questionnaire study. J. Med. Internet Res..

[25] Rutter C.M., Gatsonis C.A. (2001). A hierarchical regression approach to meta-analysis of diagnostic test accuracy evaluations. Stat. Med..

[26] Creswell J.W., Plano Clark V.L. (2011). Designing and Conducting Mixed Methods Research.

[27] Cheng Z. (2014). The Effects of Employee Involvement and Participation on Subjective Wellbeing: Evidence from Urban China. Soc. Indic. Res..

[28] Meng K. (2020). Promotion Tournament, Labor Market Tightening and Pension Generosity: A Comparative Public Policy Analysis of Pension System for Urban Workers in China (1997–2013). J. Comp. Policy Anal. Res. Pract..

[29] Huang X., Western M., Bian Y., Li Y., Côté R., Huang Y. (2019). Social networks and subjective wellbeing in Australia: New evidence from a national survey. Sociology.

[30] Bian Y. (1997). Bringing strong ties back in: Indirect ties, network bridges, and job searches in China. Am. Sociol. Rev..

[31] Centers for Disease Control and Prevention (CDC) (2021). Largest CDC COVID-19 Vaccine Effectiveness Study in Health Workers Shows mRNA Vaccines 94% Effective.

[32] Hall V., Hopkins S. (2021). COV-BOOST: Evidence to Support Rapid Booster Deployment. Lancet.

[33] Huang I., Poland G. (2021). Single-dose Oxford–AstraZeneca COVID-19 vaccine followed by a 12-week booster. Lancet.

[34] Sun S., Lin D., Operario D. (2021). Interest in COVID-19 vaccine trials participation among young adults in China: Willingness, reasons for hesitancy, and demographic and psychosocial determinants. Prev. Med. Rep..

[35] Reiter P.L., Pennell M.L., Katz M.L. (2020). Acceptability of a COVID-19 vaccine among adults in the United States: How many people would get vaccinated?. Vaccine.

[36] LÜ X. (2014). Social Policy and Regime Legitimacy: The Effects of Education Reform in China. Am. Political Sci. Rev..

[37] Burns J., Wang X. (2010). Civil Service Reform in China: Impacts on Civil Servants’ Behaviour. China Q..

[38] Chan H., Ma J. (2011). How are they paid? A study of civil service pay in China. Int. Rev. Adm. Sci..

[39] Bell S., Clarke R., Mounier-Jack S., Walker J.L., Paterson P. (2020). Parents’ and guardians’ views on the acceptability of a future COVID-19 vaccine: A multi-methods study in England. Vaccine.

[40] Nomura S., Eguchi A., Yoneoka D., Kawashima T., Tanoue Y., Murakami M., Sakamoto H., Maruyama-Sakurai K., Gilmour S., Shi S. (2021). Reasons for being unsure or unwilling regarding intention to take COVID-19 vaccine among Japanese people: A large cross-sectional national survey. Lancet Reg. Health-West. Pac..

[41] Machida M., Nakamura I., Kojima T., Saito R., Nakaya T., Hanibuchi T., Takamiya T., Odagiri Y., Fukushima N., Kikuchi H. (2021). Acceptance of a COVID-19 Vaccine in Japan during the COVID-19 Pandemic. Vaccines.

[42] Makridis C.A., Wu C. (2021). How social capital helps communities weather the COVID-19 pandemic. PLoS ONE.

[43] Lazarus J.V., Ratzan S.C., Palayew A., Gostin L.O., Larson H.J., Rabin K., Kimball S., El-Mohandes A. (2020). A global survey of potential acceptance of a COVID-19 vaccine. Nat. Med..

[44] Arghittu A., Dettori M., Dempsey E., Deiana G., Angelini C., Bechini A., Bertoni C., Boccalini S., Bonanni P., Cinquetti S. (2021). Health Communication in COVID-19 Era: Experiences from the Italian VaccinarSì Network Websites. Int. J. Environ. Res. Public Health.

